# Machine-learning classification of documented pedophilic sexual interest from eye-tracking patterns

**DOI:** 10.21203/rs.3.rs-11114979/v1

**Published:** 2026-09-29

**Authors:** David Popovic, Kirsten Jordan, Peter Fromberger, Isabel Müller, Clara Weyer, Clara Vetter, Peter Falkai, Nikolaos Koutsouleris, Norbert Nedopil, Kolja Schiltz, Jürgen Leo Müller

**Affiliations:** 1 Max Planck Institute of Psychiatry, Munich, Germany; 2 Department of Psychiatry and Psychotherapy, Ludwig-Maximilian-University, Munich, Germany; 3 Forensic Psychiatry and Psychotherapy, Department of Psychiatry and Psychotherapy, University Medical Center (UMG), Georg-August-University, Göttingen, Germany; 4 Asklepios Clinic for Forensic Psychiatry and Psychotherapy, Göttingen, Germany; 5 Munich Center for Machine Learning (MCML), Munich, Germany; 6 German Center for Mental Health (DZPG), Partner Site Munich-Augsburg, Munich, Germany; 7 Institute of Psychiatry, Psychology and Neuroscience, King’s College London, London, United Kingdom

**Keywords:** pedophilia, eye tracking, support vector machines, forensic psychiatry, machine learning

## Abstract

Assessment of pedophilic sexual interest is often embedded in crucial clinical and forensic contexts and could therefore benefit from non-invasive measures that complement clinical interviews and self-report. We investigated whether multivariate eye-tracking and behavioral patterns could classify documented pedophilic sexual interest at the individual level. The discovery sample comprised 124 adult men from five German sites, including 34 with and 90 without a documented pedophilia diagnosis according to ICD-10. Participants completed the Initial Orientation Task (IOT), Sexual Distractor Task (SDT), and Sexual Stimulus Rating task. Linear support vector machines were trained within a repeated nested cross-validation framework to predict pedophilia strictly on these eye-tracking features. The best-performing main model combined IOT and SDT, achieving 88.01% balanced accuracy, 88.24% sensitivity, 87.78% specificity, and an area under the curve of 0.93 (95% confidence interval, 0.87–0.99). Exploratory sparse modeling using only five IOT and four SDT features still yielded 87.65% balanced accuracy. This sparse model further classified all 32 participants in an independent healthy-control cohort as negative (100% specificity). Predictive features reflected relative attention to child versus adult stimuli and allocation of attention between distractors and cognitive targets. These findings support further investigation of eye-tracking measures as potential adjuncts to clinical assessment, in potentially high-stakes clinical and forensic decision-making. Prospective validation in representative cohorts containing both positive and negative cases is required to further establish generalizability and clinical utility.

## Introduction

Child sexual abuse remains one of the most devastating threats to healthy development, leaving lifelong emotional, physical, and psychological scars^1^. Global estimates suggest that as many as 12% of children are affected^1^ – a figure that underscores the urgent need for better prevention, detection, and intervention strategies^2^. Victims often suffer not only from trauma-related disorders such as post-traumatic stress disorder, depression, and substance abuse, but also from profound disruptions to their interpersonal and emotional lives^2,3^. A substantial portion of sexual offenses against children are committed by individuals who meet diagnostic criteria for pedophilia (according to ICD-10), defined by a persistent sexual preference for prepubescent children^4^. Etiological frameworks such as the Motivation-Facilitation Model^5^ and the Integrated Theory of sexual offending^6^ identify deviant sexual interest and poor self-regulation as key drivers of sexual offending and recidivism. These risk factors are now core components of forensic risk assessment and treatment programs^7–9^.

Assessment of pedophilic sexual interest commonly draws on clinical interviews, self-report, collateral information and offense history^10,11^. Each source can be informative, yet disclosure may be incomplete when shame, stigma, social desirability or legal consequences are salient. This can be especially critical in forensic contexts where the stakes are highest. To close this gap, researchers have turned to indirect and physiological approaches which aim to measure responses associated with sexual interest without relying exclusively on explicit statements^10–12^. Penile plethysmography has an established evidence base but is intrusive and can be difficult to implement^12^. Viewing-time paradigms are less invasive, and meta-analytic evidence indicates that cognitively based indirect measures provide moderate discrimination between men with and without sexual interest in children^12–14^. Eye tracking extends this approach by recording the timing and distribution of visual attention. It does not make responses immune to intentional control, but it captures behavior at a temporal resolution that may complement deliberate ratings^15^. Indeed, early studies using paradigms like the Initial Orientation Task (IOT) and Sexual Distractor Task (SDT) have shown that individuals with pedophilic interest exhibit distinct gaze patterns when exposed to sexual stimuli, with stronger discrimination for the IOT than for the SDT^16–20^. Most analyses, however, considered individual eye-movement variables or prespecified indices. Such approaches do not fully use the multivariate structure of fixation latency, duration, count and area-of-interest measures. Machine learning (ML) may help address this challenge by detecting complex, multivariate patterns across thousands of features, making it ideally suited to build predictive models from eye movement data^21–23^. In forensic psychiatry, ML has already shown promise in assessing violence risk and differentiating clinical subtypes^24^, but its application to sexual interest, particularly pedophilia, remains largely unexplored. A previous neuroimaging study applied machine learning to identify patterns associated with pedophilic offending, but eye-tracking data have not, to our knowledge, been evaluated in a comparable multivariate framework^25^.

In this proof-of-concept study, we combined supervised ML with eye-tracking and behavioral data from three experimental paradigms: the IOT, the SDT and a Sexual Stimulus Rating task (SSR), each targeting different aspects of sexual interest and attentional control. We sought to (1) compare domain-specific and stacked classifiers for a documented ICD-10: F65.4 diagnosis; (2) describe the eye-movement features that contributed most consistently to classification; (3) examine specificity in an independent, negative-only healthy-control sample; and (4) explore model performance across clinical and forensic subgroups and associations with emotional responses during task exposure. We expected the two eye-tracking tasks to provide complementary information about age-related sexual interest and attentional allocation. The intended use at this stage is research evaluation of an adjunctive measure, not standalone diagnosis or prediction of offending.

## Methods

This study followed the TRIPOD+AI guidelines for reporting clinical prediction models that use regression or ML methods (Supplementary Table S1)^26,27^.

### Study Participants

This study is part of a larger project to measure sexual interest based on eye movements. Previous analyses based on univariate methods, selected data and partly smaller samples have already been published^16,18,28–33^. Participants were recruited between 2010 and 2020 across five German sites: Uchtspringe, Bremen, Moringen (forensic inpatients), Göttingen (controls: discovery and independent specificity sample, outpatients, forensic inpatients), Hannoversch Münden (controls). Exclusion criteria for all participants were acute psychotic episodes or substance abuse during the previous month, and inability or refusal to provide informed consent. All data for inpatients were obtained from forensic records. Sexual orientation with respect to gender was assessed with the Kinsey-Scale^34^. Intelligence (IQ) was measured using the Wechsler Adult Intelligence Scale^35^. All participants provided written informed consent in accordance with the Declaration of Helsinki. The study received a positive vote by the Ethics Committee of the Medical Faculty of the Georg-August-University of Göttingen. The pedophilic group (n = 34; ICD-10: F65.4) included 26 forensic inpatients with documented child sexual offenses (CSO) and 8 outpatients with self-reported CSO. The non-pedophilic group (n = 90) comprised 6 forensic inpatients with documented CSO, 18 outpatients with self-reported CSO, 14 forensic inpatients with sexual and other offenses, and 52 healthy controls with no offense history. An independent specificity sample included 32 additional non-pedophilic healthy male controls^36,37^ (Table 1, Supplementary Information S1, S2).

### Experimental Procedures

Sexual stimuli were drawn from non-pornographic, unclothed images in the Not-Real-People (NRP) set^38^, categorized as prepubescent “boy” or “girl” (Tanner stages 1–2) and postpubescent “man” or “woman” (Tanner stages 4–5)^39^, with 16 images per category. Cognitive stimuli in the SDT consisted of 64 three-dimensional mental rotation figure pairs^40^ (Figure 1, Supplementary Information S2). The IOT presented two sexual images – one adult, one child of the same gender – and measures fixation latency, and fixation time to assess automatic, and controlled attention allocation to sexually salient features^39^. The SDT assessed sexual interest and attentional control by requiring participants to complete a mental rotation task while sexually relevant or non-relevant distractors are shown; task interference was quantified through gaze behavior, i.e., the eye movement towards the distractor and the stimulus^18,30–32^. In the SSR, participants rated all images for arousal and valence on a 9-point scale; viewing time was covertly recorded^16,30^. In a subsample (n=47), emotional responses before and after each task were assessed using the self-developed Current State of Emotions Questionnaire (CSEQ), which assesses eight emotion domains (i.e., rage, happiness, disgust, cheerfulness, anxiety, sadness, arousal, anger) on a 9-point Likert scale^33,41^.

### Eye-tracking Data Acquisition and Preprocessing

Eye movements were recorded using an SMI iView X RED system and analyzed with BeGaze 3 software (SensoMotoric Instruments GmbH, Berlin, Germany). Fixations were mapped onto predefined areas of interest (AOIs), including full-body regions (man, woman, boy, girl) and subregions (head, chest, abdomen, genitals). Only AOIs matching each participant’s sexual preference (based on the Kinsey score or victim profile) were analyzed, resulting in “child” and “adult” stimulus categories. For SDT, fixations toward sexual distractors were compared to those toward mental rotation targets to assess attentional control^42^. We computed a Child Preference Index (CPI) and an Attentional Control Index (ACI) as ratio scores indicating preference for child stimuli and inhibitory control, respectively. This yielded 134 IOT, 301 SDT, and 12 SSR features for ML analyses (Supplementary Information S3, Supplementary Tables S2-S5).

### Machine Learning Pipeline

We used the NeuroMiner platform (v1.4, http://proniapredictors.eu/neurominer/index.html,^42^) to train linear support vector machine (SVM) classifiers^21^ on the IOT, SDT, and SSR domains separately, to distinguish pedophilic from non-pedophilic individuals. Models were embedded in a repeated 10×10-fold nested cross-validation framework for hyperparameter tuning and out-of-sample testing^43^. Balanced accuracy (BAC) was the optimization criterion. Class imbalances were handled via SVM hyperplane weighting. MiTo develop a clinically scalable tool, feature selection for IOT and SDT was performed using greedy forward wrappers^44^ to generate sparse models with the most predictive features. All features were scaled and corrected for age, IQ, and hospitalization based on the control subpopulation within the cross-validation structure using partial correlations^44^. Sign-based consistency (SBC)^45^ and cross-validation ratios (CVR)^44^ were used to identify robust predictors, which were then used to retrain sparse models and create stacked models. Stacked models combined cross-validated predictions from multiple base classifiers (e.g., sparse IOT, SDT, and SSR models) as inputs to a meta-learner that learns optimal weights, yielding a single, stacked classifier^46^. These models were then validated in an independent control sample (n = 32) and compared against 5000 label-shuffled permutations^46^. Furthermore, performance of the best-performing model was compared across subgroups defined by victim sex, hospitalization status, crime and conviction history, pedophilic interest, ICD-10 diagnoses, and pharmacological treatment. In a subsample for which CSEQ data were available (n = 47), decision scores were correlated with pre-task, post-task, and pre-to-post CSEQ change scores (Supplementary Information S4–8).

## Results

### Group-Level Differences at Baseline

The discovery sample comprised 124 adult men, including 34 individuals with and 90 without a documented pedophilia diagnosis (ICD-10: F65.4). Compared with non-pedophilic individuals, pedophilic individuals were older [mean (standard deviation), 40.18 (10.28) vs. 32.10 (12.48) years; P=4.84E-04] and had a lower IQ [84.06 (19.48) vs. 104.79 (20.93); P=1.58E-05]. They were also less frequently classified as heterosexual [44.12 % vs. 75.56 %; P=2.12E-03] and more frequently as bisexual [32.35 % vs. 2.22 %; P=5.67E-06] with regards to their teleophilic interest. However, frequency of teleiophilic homosexual orientation, Kinsey scores, age at first sexual intercourse, and duration of the longest partnership did not differ. Sexual fantasies involving minors and reported acting on such fantasies were more frequent among pedophilic individuals [73.53 % vs. 16.67 %; P=1.67E-08] (Table 1). The groups also differed in clinical and forensic composition. Most pedophilic individuals were forensic inpatients with a history of child sexual offenses, had longer inpatient stays, and more frequently had additional personality disorders (ICD-10: F60–F69) or neurodevelopmental disorders originating in childhood or adolescence (ICD-10: F90–F99).

Among the 72 participants with self-reported or convicted offenses, pedophilic individuals had more victims [8.56 (16.97) vs. 0.79 (2.05); P=7.73E-13] and younger victims [9.09 (2.70) versus 14.07 (6.98) years; P=0.02]. Sexual and child sexual offense histories and convictions for child sexual abuse were also more frequent, whereas having no previous conviction was more common among non-pedophilic individuals. The number of previous convictions, age at the previous offense, and index-offense sentence length did not differ between groups (Supplementary Table S6).

The independent specificity cohort comprised 32 non-pedophilic controls and was compared with the 52 controls in the discovery sample. The samples did not differ in age, IQ, sexual-orientation distribution, or Kinsey scores (Supplementary Table S7). Finally, in the subsample of 47 participants, which had completed the CSEQ during the eye-tracking tasks, no group-level differences were observed between pedophilic and non-pedophilic individuals with regards to their emotional states before or after the IOT, SDT, or SSR experiments, or in their pre-to-post experiment changes (Supplementary Table S8).

### Model Performance in Discovery and Independent Specificity Sample

The IOT model correctly classified 28 of 34 participants with a pedophilia diagnosis and 78 of 90 participants without a pedophilia diagnosis, corresponding to a sensitivity of 82.35%, specificity of 86.67%, and a BAC of 84.51% (Figure 2; Table 2, Supplementary Table S9). The SDT and SSR models yielded BACs of 68.14% and 75.65%, respectively. Stacked models reached BACs of 88.01% (IOT+SDT), 83.59% (IOT+SSR), 75.49% (SDT+SSR), and 85.98% (IOT+SDT+SSR). Sparse models based on the most robust predictors (5 IOT, 4 SDT, and 3 SSR features; Figure 2) improved performance, yielding BACs of 87.09% for IOT, 71.11% for SDT, and 80.62% for SSR. Stacked sparse models achieved BACs of 87.65% (IOT+SDT), 86.54% (IOT+SSR), 81.93% (SDT+SSR), and 85.07% (IOT+SDT+SSR). When applied to the independent specificity sample (n = 32), sparse IOT, SDT, and SSR models misclassified 2, 5, and 3 individuals, yielding specificities of 93.75%, 84.38%, and 90.63%, respectively. All IOT-containing stacked models achieved 100% specificity, while SDT+SSR reached 87.50% (Table 2, Supplementary Table S9).

### Model Performance Across Clinical and Forensic Subgroups

Performance of the stacked IOT+SDT classifier was broadly consistent across clinical and forensic subgroups, although heterogeneity emerged for offense history (χ2=15.77; P=.015) and personality disorder diagnosis (χ2=5.72; P=.017); Table 3). BAC was 77.96% among participants with any sexual-offense history, 73.43% among those with sexual offenses against children, 65.91% among those with a child-pornography offense history, and 58.33% among those with offenses against adults and children. In the child-sexual-offense subgroup, reduced performance mainly reflected low specificity (54.55%) despite high sensitivity (92.31%), whereas the subgroup with offenses against adults and children showed lower sensitivity (66.67%) and specificity (50.00%). Participants without a sexual-offense or child-pornography history and those without any criminal offense were almost uniformly classified as non-pedophilic, with specificities of 98.31% and 98.11%, respectively. However, these groups contained no pedophilic cases, preventing calculation of sensitivity and balanced accuracy. We did not observe any clear evidence of heterogeneity by victim sex, hospitalization status, or conviction history. Notably, the performance of the IOT+SDT model did not differ with regards to reported sexual fantasies involving children, acting on such fantasies, or denial of pedophilic interest.

### Predictive eye-movement patterns

Wrapper-based feature selection identified five of 134 IOT features as significant, based on CVR and SBC mapping (Figure 2A, B; Table 2; Supplementary Tables S10-S12). These included AOI entries, fixation durations, and fixation counts, particularly for head and abdomen regions, and were related to the CPI. While pedophilic individuals showed similar viewing patterns for child and adult stimuli, they fixated on child stimuli more frequently and for longer durations than controls, who clearly preferred adult stimuli. In the SDT model, four of 301 features were significant (Figure 2C/D). Three predicted pedophilic interest and reflected reduced attentional control: early fixations on adult distractors (especially the genital region), and earlier fixations on child distractors followed by delayed focus on the respective mental rotation figures. One feature was predictive of non-pedophilic individuals, who more often fixated early and exclusively on the mental rotation figures rather than on child distractors. In the SSR model, three of 12 features were predictive: sexual arousal ratings, and CPI-viewing time. Pedophilic individuals rated child stimuli as more arousing and adult stimuli as less arousing compared to controls and viewed child stimuli for longer durations (Figure 2E/F). Across all stacked models, the IOT consistently contributed the strongest predictive weights (Figure 3A).

### Association Between Model Decision Scores and Emotional States

In an exploratory manner, decision scores from the retrained sparse models were correlated with pre-task, post-task, and pre-to-post change scores on the CSEQ in a subsample (n = 47) (Figure 3B/C; Supplementary Table S13). For the IOT model, higher scores correlated with pre-to-post increases of sadness (ρ = 0.37, P = .016), anger (ρ = 0.37, P = .006), and decreases happiness (ρ = −0.33, P = .033). SDT scores were linked to pre-task rage (ρ = 0.31, P = .042), pre-task (ρ = 0.34, P = .024) and post-task (ρ = 0.30, P = .048) anger. SSR scores correlated with post-task sexual arousal (ρ = 0.40, P = .009) and pre-to-post increases in arousal (ρ = 0.32, P = .035). No group-level differences in CSEQ scores were observed between pedophilic and non-pedophilic participants.

## Discussion

In this study, we present the first application of supervised ML to eye-tracking and behavioral data from three experimental paradigms (IOT, SDT, SSR) to predict pedophilic sexual interest. Our findings extend earlier univariate work on initial orientation, viewing time and sexual distraction by showing that several small, interrelated features can be combined at the individual level^47^. Model performance remained robust across diverse clinical and forensic subgroups, and the best-performing model was further validated in an independent sample of non-pedophilic individuals. Additionally, we explored associations between model decision scores and participants’ self-reported emotional states, offering insight into the affective dynamics underlying deviant sexual interest.

All three models (IOT, SDT, SSR) successfully distinguished pedophilic from non-pedophilic individuals with moderate to high accuracy. The strongest performance emerged from stacking the two eye-tracking models (IOT+SDT), which consistently outperformed both individual models and those involving behavioral ratings (SSR). Strikingly, even sparse models – using just a handful of predictive features – maintained high accuracy, particularly when applied to a independent specificity sample of healthy controls. These findings not only replicate earlier univariate work^48^, but also extend it by identifying multivariate eye-tracking patterns predictive of pedophilic interest captured through ML. Importantly, the superior performance of eye-tracking-based models (IOT, SDT) over behavioral-only approaches (SSR) suggests that gaze dynamics may provide a more sensitive and potentially less manipulable marker of pedophilic interest – further highlighting the unique potential of eye-tracking in this context.

In both the IOT and SSR tasks, pedophilic individuals showed comparable gaze patterns toward child and adult stimuli – unlike non-pedophilic participants, who clearly preferred adult stimuli. It was not absolute fixation behavior but relative patterns – captured by “child preference indices” – that most reliably distinguished the groups. Pedophilic individuals viewed child stimuli more often and for longer durations, and rated them as more sexually arousing than non-pedophilic individuals, who showed the reverse pattern. This agrees with prior evidence that pedophilic sexual interest can involve both greater response to children and lower sexual interest in adults^47,48^. For the SDT, key predictive features reflected reduced attentional control: pedophilic individuals fixated earlier and more frequently on child distractors than on task-relevant mental rotation figures, indicating weaker inhibition. In some cases, this pattern also applied to adult stimuli. These findings echo prior studies reporting impaired attentional control in individuals with child sexual offenses^48^. Compared with univariate approaches, our multivariate models identified a broader set of interrelated eye-movement features, including fixation counts, durations, and AOI entry patterns, suggesting that pedophilic interest may be better characterized by multivariate patterns of eye-movement behavior than by any single measure alone. Gaze toward specific regions, such as the head and abdomen, was more informative than whole-image metrics. Our findings suggest that a small, targeted set of eye-tracking features, capturing both deviant sexual preference (IOT) and diminished attentional control (SDT), can robustly identify pedophilic interest. The stacked IOT+SDT model, but not the addition of the behavioral SSR task, yielded the best classification performance, suggesting that this combined eye-movement pattern represents the best characterization of pedophilic interest. This pattern may align with established risk factors for sexual offending, including impaired self-regulation and persistent attraction to children^9,49^. The dominance of the IOT in stacked models suggests that age-related preference signals were more stable than the interference signal in this sample. The failure of SSR to add performance may reflect the greater role of deliberate response, overlapping information, or measurement noise rather than an absence of clinical relevance.

The performance of the best-performing model was largely consistent across clinical and forensic subgroups, differing only by offense history and personality disorder status. This suggests that the model captures pedophilic interest relatively independently of most clinical, forensic, and treatment-related characteristics, while indicating that offense-related factors and personality pathology may affect classification in some cases. Notably, this also applied to subgroups defined by whether individuals reported, acted on, or denied having sexual fantasies involving children. This supports the notion that self-reports alone are insufficient for reliably assessing pedophilic interest, a limitation well known in forensic contexts^50^. As proposed by Spiering and Everaerd^51^, a comprehensive assessment of sexual interest should integrate explicit cognitions (e.g., fantasies), implicit processes, and physiological arousal. Our findings reinforce the value of indirect, objective measures, such as eye-tracking, for improving the assessment and diagnosis of pedophilia^19^.

Model predictions were further contextualized by their associations with emotional responses, linking higher classifier scores to a pattern of increased negative affect and arousal, primarily reflected in pre-to-post task changes. This pattern suggests that emotional dysregulation, particularly heightened negative affect and arousal in response to sexual stimuli, was associated with a stronger model signal for pedophilic interest. These findings align with prior research highlighting deficits in emotion regulation as key risk factors for sexual and violent offending^48,52^. It is possible that some individuals experienced strong arousal during the tasks but lacked the regulatory capacity to manage or suppress it, potentially exposing mechanisms that underlie future offending risk. Alternatively, pedophilic individuals might have experienced sadness, anger and reduced happiness due to possible stigmatization^53^.

### Limitations

A key limitation of this study is that experimentally measured sexual interest reflects momentary responses and should not be equated with a clinical diagnosis of pedophilia or pedophilic disorder. As such, our ML tools are intended to support – not replace – clinical assessment, particularly in cases involving denial, social desirability, or faking. Additionally, our sample size is modest for ML applications, which may limit generalizability and risk overfitting. Given the high stigma surrounding pedophilia^54,55^, false positives could carry serious consequences. While our models demonstrated high specificity in an independent control sample, larger, more diverse multicenter cohorts are needed to validate their clinical utility. Finally, although rare, we cannot fully exclude the possibility that exposure to sexual stimuli could increase distress or even risk in vulnerable individuals. Any future application of this paradigm must carefully balance the scientific value against the ethical responsibility to minimize harm^54^.

## Conclusion

This study is, to our knowledge, the first to demonstrate that a sparse set of eye-tracking and behavioral features captured during controlled experimental tasks can be leveraged by ML to robustly classify pedophilic interest across forensic and clinical subgroups. Notably, the most predictive signals arose not from explicit ratings or self-report, but from subtle gaze patterns and attentional dynamics; features that are potentially harder to fake. By applying a multivariate approach, we extend previous univariate findings and show that a specific eye movement pattern from two eye-tracking tasks provides the best classification performance. Our findings point toward the potential of eye-tracking data for individual-level, data-driven classification of pedophilic interest. Further studies should compare the performance of such multivariate approaches with previous univariate analyses at the individual level. It has been suggested that indirect sexual interest measures should be associated with independent indicators of deviant sexual interest, such as criminological variables, in order to confirm their validity^13^. As ML offers the possibility to analyze large amounts of variables together to look for specific patterns, future studies should integrate criminological variables, e.g., number of victims and Screening Scale for Pedophilic Interests scores^56,57^, but also clinical parameters to better characterize pedophilic and non-pedophilic individuals. Although our models are not diagnostic tools, they offer a glimpse into a future where sexual interest assessments are grounded in objective, quantifiable evidence rather than disclosure or suspicion. With further validation in larger and more diverse samples, this approach could inform a new generation of forensic tools – tools that are less vulnerable to deception, more precise in risk assessment, and potentially transformative for both prevention and treatment.

## Supplementary Material

Supplementary Files

This is a list of supplementary files associated with this preprint. Click to download.

- SupplementTP.docx
- GraphicalAbstract.png

## Figures and Tables

**Figure 1:**
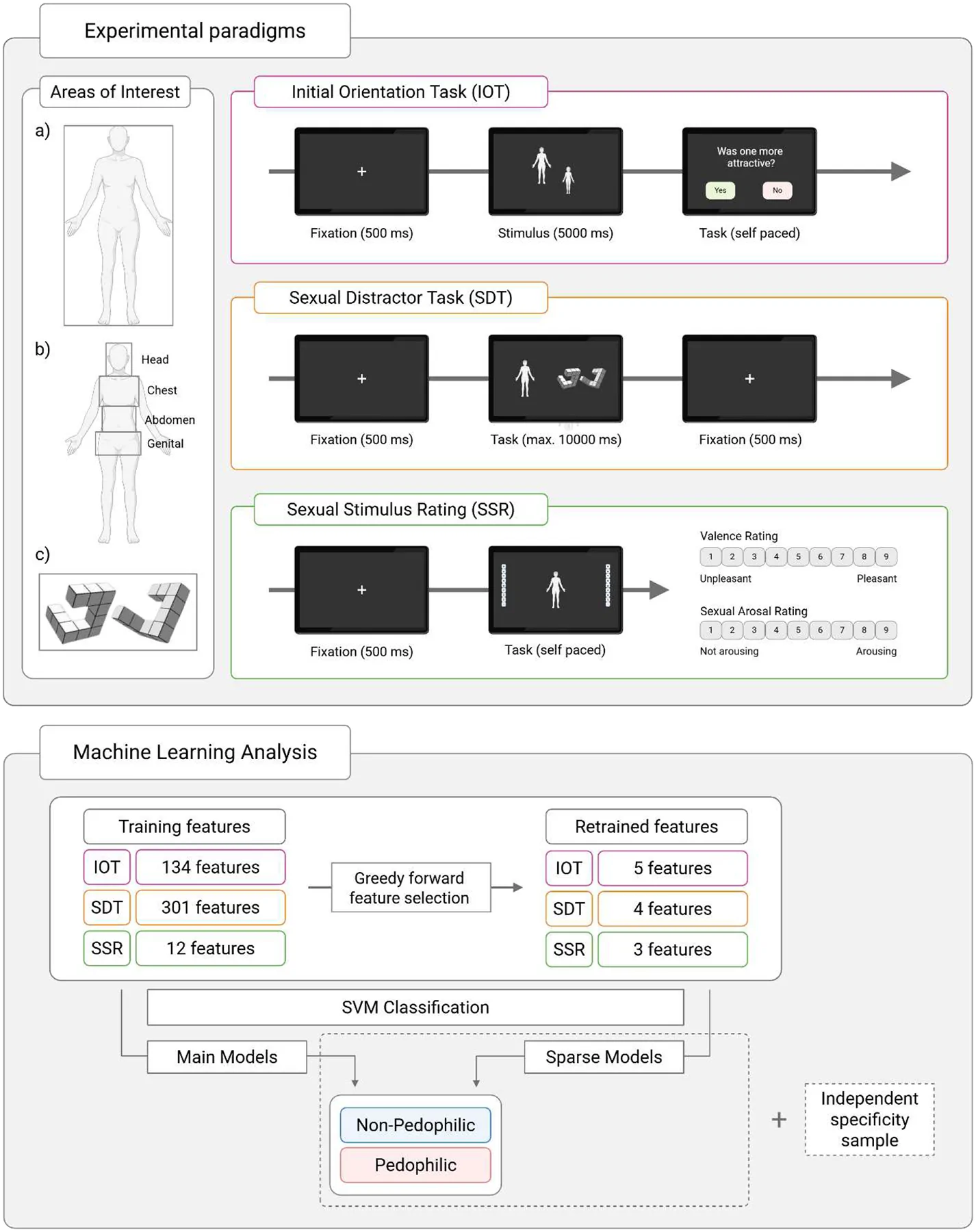
Experimental Paradigms and Machine Learning Workflow **Upper panel:** Areas of interest and schematic overview of the three experimental paradigms. Each task included an initial fixation period. In the Initial Orientation Task (IOT), participants viewed two stimuli, one adult and one child of the same gender, and indicated which stimulus was more attractive. In the Sexual Distractor Task (SDT), participants completed a mental-rotation task while sexually relevant or irrelevant distractors were presented. In the Sexual Stimulus Rating task (SSR), participants rated all images on 9-point scales for valence and sexual arousal. **Lower panel:** Machine learning workflow. Support vector machine (SVM) classifiers were trained using the full feature set from IOT (134 features), SDT (301 features) and SSR (12 features). Wrapper-based greedy forward feature selection was then used to derive sparse retrained models based on a reduced feature set from IOT (5 features), SDT (4 features) and SSR (3 features). Sparse models were additionally examined in an independent specificity sample comprising non-pedophilic individuals. *Abbreviations*: IOT, Initial Orientation Task; SDT, Sexual Distractor Task; SSR, Sexual Stimulus Rating task; SVM, support vector machine. Created in BioRender. Weyer, C., Popovic, D. (2026) https://BioRender.com/dstgi65

**Figure 2:**
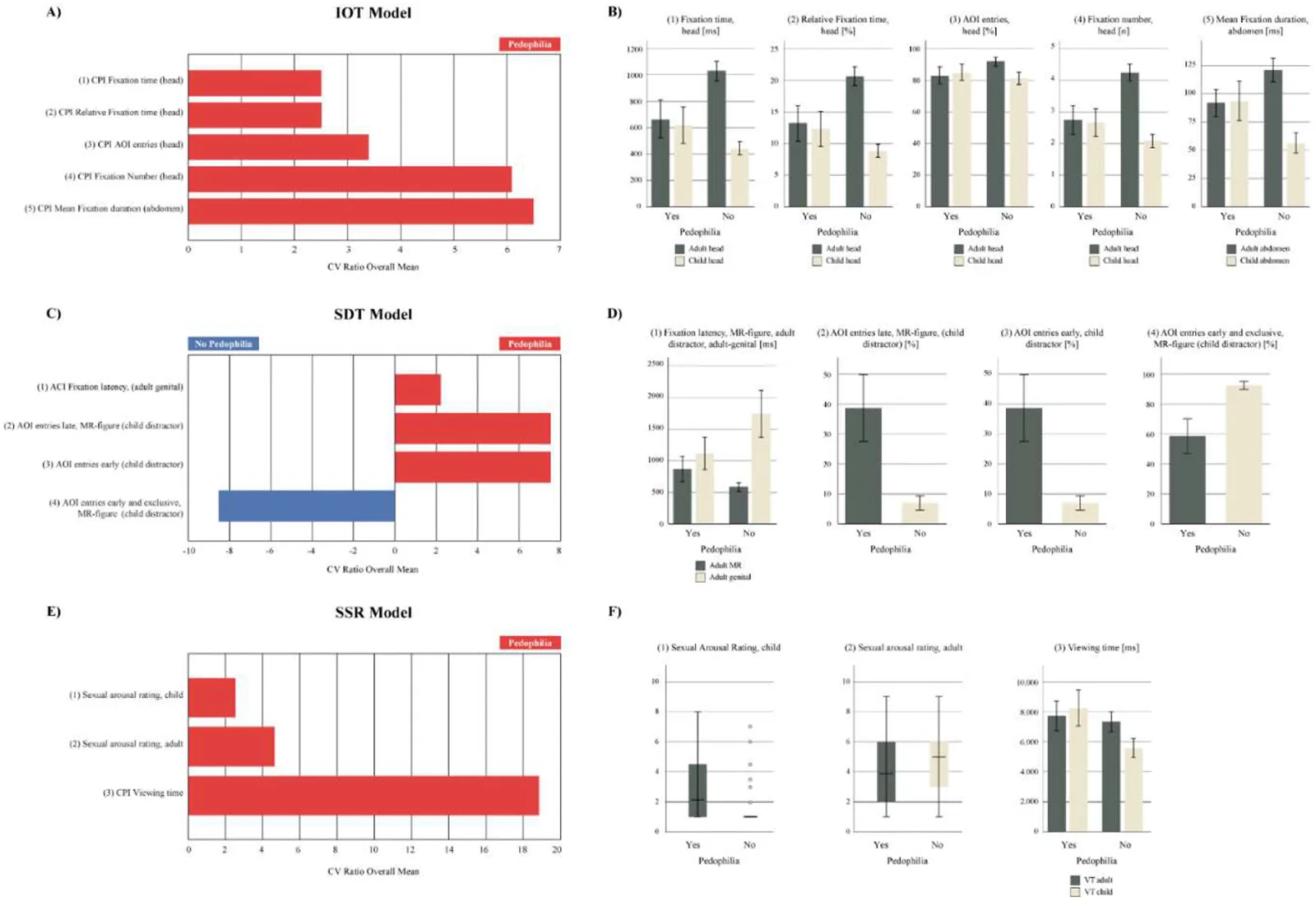
Visualization of the main and sparse pedophilia models and significant input features. **(A,C,E)** Mean cross-validation ratios (CVRs) of feature weights for the significant input features of the sparse retrained models are shown for the IOT (A), SDT (C) and SSR (E). Positive CVRs, predictive of pedophilic individuals, are displayed in red, and negative CVRs, predictive of healthy controls, are displayed in blue. **(B, D, F)** Group differences in the significant input features are shown for pedophilic and non-pedophilic individuals, and, where applicable, separately for child and adult areas of interest. If indices such as the child preference index (CPI) or attention control index (ACI) are shown in the left panels (A, C, E), the corresponding raw measures used to compute these indices are presented in the right panels (B, D, F). In panel F1, horizontal bars indicate medians, and individual points indicate outliers relative to the sample distribution. *Abbreviations:* ACI, attentional control index; AOI, area of interest; CPI, child preference index; CVR, cross-validation ratio; IOT, Initial Orientation Task; SDT, Sexual Distractor Task; SSR, Sexual Stimulus Rating task.

**Figure 3:**
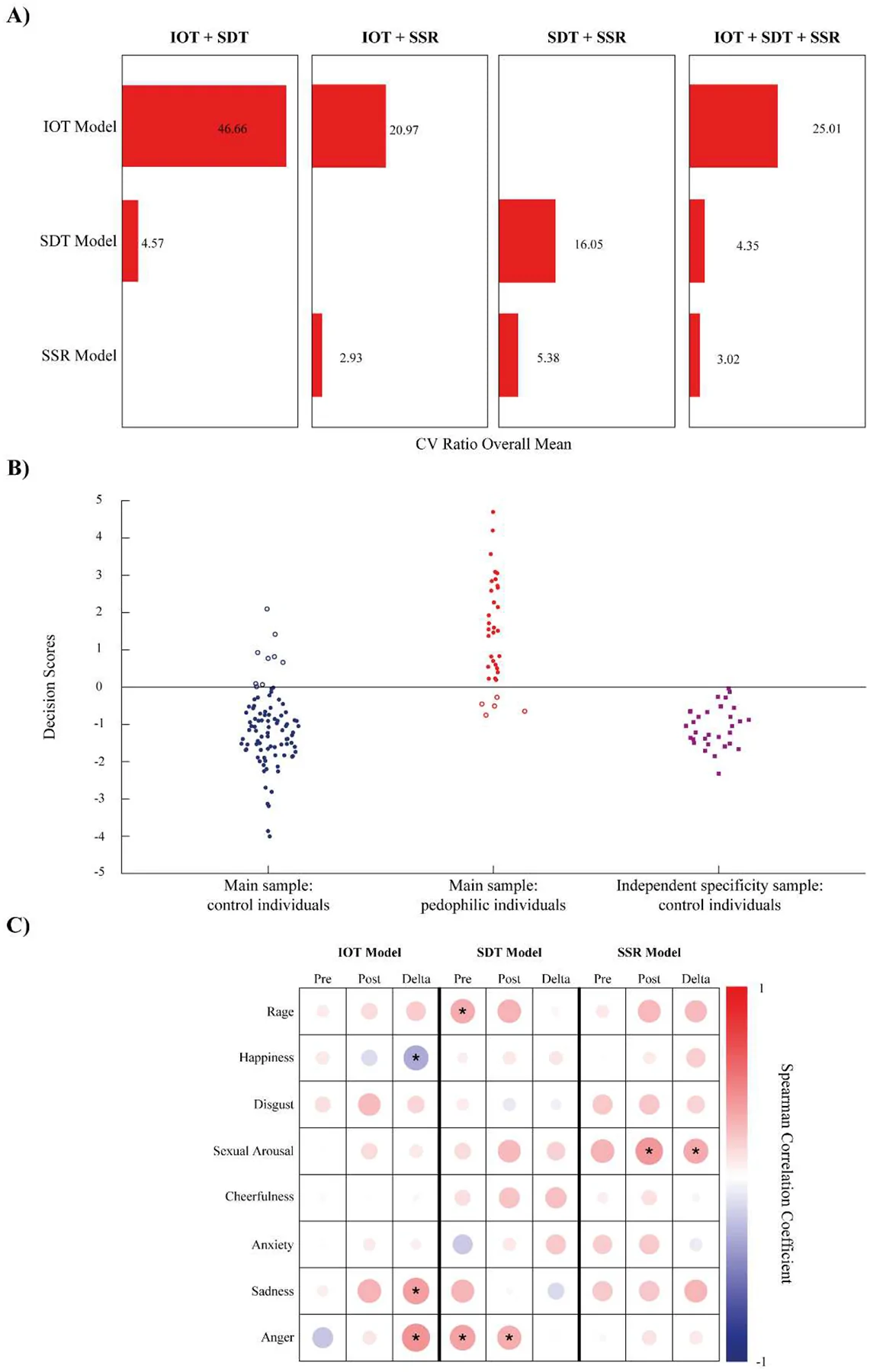
Visualization, performance and interaction with emotional states of selected stacked models. **(A)** Overall mean cross-validation ratios (CVRs) of the feature weights for the stacked IOT+SDT, IOT+SSR, SDT+SSR and IOT+SDT+SSR models. Positive CVRs, predictive of pedophilic individuals, are displayed in red. **(B)** Decision scores from the IOT+SDT model for the healthy controls and the pedophilic individuals from the main study sample, as well as healthy controls in the independent specificity sample. Scores above the decision boundary (>0) indicate that the IOT+SDT model classified these individuals as pedophilic, while scores below the decision boundary (<0) indicate that the IOT+SDT model classified these individuals as healthy controls. Open circles denote misclassified individuals. **(C)** Spearman correlations between the decision scores of the IOT, SDT and SSR models and eight self-rated emotional states before (pre), after (post), and as pre-to-post task change scores (delta) for the respective experimental task. Delta was calculated as post minus pre, where positive values reflect an increase and negative values a decrease in the respective emotional state. Red indicates positive correlations, while blue colors reflect negative correlations. Furthermore, larger circles correspond to stronger and smaller circles to weaker correlations. Significant correlations are marked with an asterisk. *Abbreviations:* CV, Cross-Validation; IOT, Initial Orientation Task; SDT, Sexual Distractor Task; SSR, Sexual Stimulus Rating Task

**Table 1:** Clinical and sociodemographic characteristics of the main study sample

	Full Sample	Pedophilic Individuals	Non-Pedophilic Individuals	U/ χ^2^	P_FDR_
N	124	34	90		
Age	34.31 (12.41)	40.18 (10.28)	32.1 (12.48)	3.75	**4.84E-04**
IQ	99.18 (22.46)	84.06 (19.48)	104.79 (20.93)	−4.62	**1.58E-05**
Sexual Orientation					
Heterosexual	66.94 %	44.12 %	75.56 %	11.02	**2.12E-03**
Homosexual	22.58 %	23.53 %	22.22 %	0.02	0.88
Bisexual	10.48 %	32.35 %	2.22 %	23.87	**5.67E-06**
Kinsey Score	1.64 (2.32)	2.18 (2.18)	1.43 (2.35)	1.99	0.09
Age at first sexual intercourse (years)	17.73 (4.83)	16.97 (5.43)	17.99 (4.62)	−1.18	0.36
Duration of longest partnership (months)	61.05 (86.04)	62.23 (62.6)	60.65 (93)	0.87	0.48
Sexual fantasies with minors	32.26 %	73.53 %	16.67 %	36.51	**1.67E-08**
Acting out of sexual fantasies with minors	19.35 %	55.88 %	5.56 %	40.04	**4.09E-09**
Subgroup					
CSO, Inpatients (n=32)	25.81 %	76.47 % (n=26)	6.67 % (n=6)	62.80	**7.56E-14**
CSO, Outpatients, (n=26)	20.97 %	23.53 % (n=8)	20 % (n=18)	0.19	0.71
Non-CSO, Inpatients, (n=14)	11.29 %	0 % (n=0)	15.56 % (n=14)	5.96	**0.02**
Healthy controls (n=52)	41.94 %	0 % (n=0)	57.78 % (n=52)	33.83	**4.96E-08**
Duration of inpatient stay (months)	46.53 (81.48)	98.06 (80.33)	27.07 (73.38)	5.70	**8.05E-08**
ICD-10 Diagnosis					
F00-F09	1.61 %	0 %	2.22 %	0.77	0.48
F10-F19	20.97 %	32.35 %	16.67 %	3.66	0.10
F20-F29	6.45 %	8.82 %	5.56 %	0.44	0.62
F30-F39	8.06 %	14.71 %	5.56 %	2.79	0.16
F40-F49	4.84 %	2.94 %	5.56 %	0.37	0.63
F50-F59	4.84 %	2.94 %	5.56 %	0.37	0.63
F60-F69	35.48 %	61.76 %	25.56 %	14.13	**4.84E-04**
F70-F79	8.06 %	11.76 %	6.67 %	0.87	0.48
F80-F89	0.81 %	2.94 %	0 %	2.67	0.16
F90-F99	2.42 %	8.82 %	0 %	8.14	**0.01**

Results are reported as mean (SD) for continuous variables and as relative frequencies for categorical variables. Group differences were assessed using the nonparametric Mann-Whitney U test for continuous variables and the χ^2^ test for dichotomous variables. All P values were corrected for multiple comparisons using the false discovery rate (FDR)^58^ procedure at a significance threshold of q=0.05. Significant P values are highlighted in bold. The ICD-10 category F60-F69 does not include pedophilia (F65.4). *Abbreviations:* CSO, child sexual offense; IQ, intelligence quotient.

**Table 2:** Performance metrics of the prediction models in the main study cohort and the independent specificity sample.

	T	TP	FN	TN	FP	SENS	SPEC	PPV	NPV	AUC (95%CI)	BAC	PSI	NNP	NND	Pfdr
**Main Models**															
IOT	124	28	6	78	12	82.35	86.67	70.00	92.86	0.93 (0.86–0.99)	84.51	62.86	1.59	1.45	<2.0E-04
SDT	124	18	16	75	15	52.94	83.33	54.55	82.42	0.78 (0.68–0.88)	68.14	36.96	2.71	2.76	<2.0E-04
SSR	124	25	9	70	20	73.53	77.78	55.56	88.61	0.82 (0.73–0.91)	75.65	44.16	2.26	1.95	<2.0E-04
**IOT+SDT**	124	**30**	**4**	**79**	**11**	**88.24**	**87.78**	**73.17**	**95.18**	**0.93 (0.87–0.99)**	**88.01**	**68.35**	**1.46**	**1.32**	**<2.0E-04**
IOT+SSR	124	27	7	79	11	79.41	87.78	71.05	91.86	0.92 (0.85–0.98)	83.59	62.91	1.59	1.49	<2.0E-04
SDT+SSR	124	23	11	75	15	67.65	83.33	60.53	87.21	0.86 (0.77–0.94)	75.49	47.74	2.09	1.96	<2.0E-04
IOT+SDT+SSR	124	29	5	78	12	85.29	86.67	70.73	93.98	0.92 (0.86–0.99)	85.98	64.71	1.55	1.39	<2.0E-04
**Sparse Retrained Models**															
IOT	124	29	5	80	10	85.29	88.89	74.36	94.12	0.94 (0.88–1.00)	87.09	68.48	1.46	1.35	<2.0E-04
SDT	124	17	17	83	7	50.00	92.22	70.83	83.00	0.78 (0.68–0.88)	71.11	53.83	1.86	2.37	<2.0E-04
SSR	124	28	6	71	19	82.35	78.89	59.57	92.21	0.86 (0.78–0.95)	80.62	51.78	1.93	1.63	<2.0E-04
**IOT+SDT**	124	**29**	**5**	**81**	**9**	**85.29**	**90.00**	**76.32**	**94.19**	**0.95 (0.90–1.00)**	**87.65**	**70.50**	**1.42**	**1.33**	**<2.0E-04**
IOT+SSR	124	29	5	79	11	85.29	87.78	72.50	94.05	0.94 (0.88–1.00)	86.54	66.55	1.50	1.37	<2.0E-04
SDT+SSR	124	27	7	76	14	79.41	84.44	65.85	91.57	0.90 (0.83–0.97)	81.93	57.42	1.74	1.57	<2.0E-04
IOT+SDT+SSR	124	28	6	79	11	82.35	87.78	71.79	92.94	0.95 (0.90–1.00)	85.07	64.74	1.54	1.43	<2.0E-04
**Independent Specificity Sample**															
IOT	32	0	0	30	2		93.75	0.00	100.00			0.00			<2.0E-04
SDT	32	0	0	27	5		84.38	0.00	100.00			0.00			<2.0E-04
SSR	32	0	0	29	3		90.63	0.00	100.00			0.00			<2.0E-04
**IOT+SDT**	32	**0**	**0**	**32**	**0**		**100.00**		**100.00**						<2.0E-04
IOT+SSR	32	0	0	32	0		100.00		100.00						<2.0E-04
SDT+SSR	32	0	0	28	4		87.50	0.00	100.00			0.00			<2.0E-04
IOT+SDT+SSR	32	0	0	32	0		100.00		100.00						<2.0E-04

Performance of the prediction models in the discovery and the independent specificity sample. Main models were based on the full feature sets. Sparse retrained models were restricted to the most robust predictors. As the independent specificity sample included only non-pedophilic healthy controls (HC), only specificity-based performance indices are reported for that sample. Group-level differences were assessed using the nonparametric Mann-Whitney-U-Test. P values were calculated by comparing the BAC of each model against a null distribution of 5000 models trained on random permutations of the target labels and were corrected for multiple comparisons using the false discovery rate (FDR) procedure at a significance threshold of q=0.05^58^. The best-performing model (i.e., IOT+SDT) and significant P values are highlighted in bold. *Abbreviations:* AUC, area under the curve; BAC, balanced accuracy; FN, number of false negatives; FP, number of false positives; IOT, Initial Orientation Task; NND, number needed to diagnose; NNP, number needed to predict; NPV, negative predictive value; PPV, positive predictive value; PSI, prognostic summary index; SDT, Sexual Distractor Task; Sens, sensitivity; Spec, specificity; SSR, Sexual Stimulus Rating; T, Total cases; TN, number of true negatives; TP, number of true positives.

**Table 3:** Performance of the sparse IOT+SDT model across clinical and forensic subgroups.

	T	TP	FN	TN	FP	SENS	SPEC	PPV	NPV	BAC	PSI	NNP	NND	χ^2^	P
**Victim Sex**															
Male + Female	12	7	1	2	2	87.50%	50.00%	77.78%	66.67%	68.75%	44.44	2.25	2.67	0.55	0.46
Female	80	12	3	60	5	80.00%	92.31%	70.59%	95.24%	86.15%	65.83	1.52	1.38		
Male	32	10	1	19	2	90.91%	90.48%	83.33%	95.00%	90.69%	78.33	1.28	1.23		
**Hospitalization Status**															
Hospitalized	46	24	2	15	5	92.31%	75.00%	82.76%	88.24%	83.65%	70.99	1.41	1.49	1.13	0.29
Not Hospitalized	78	5	3	66	4	62.50%	94.29%	55.56%	95.65%	78.39%	51.21	1.95	1.76		
**Offense History**															
Sexual offense	51	28	4	13	6	87.50%	68.42%	82.35%	76.47%	77.96%	58.82	1.70	1.79		
Sexual offense against adults	6	0	0	6	0		100.00%		100.00%		NA	NA	NA	15.77	**0.015**
Sexual offense against children	37	24	2	6	5	92.31%	54.55%	82.76%	75.00%	73.43%	57.76	1.73	2.13		
Sexual offense against adults and children	8	4	2	1	1	66.67%	50.00%	80.00%	33.33%	58.33%	13.33	7.50	6.00		
Child pornography	13	1	1	9	2	50.00%	81.82%	33.33%	90.00%	65.91%	23.33	4.29	3.14		
Assault	3	0	0	3	0		100.00%		100.00%		NA	NA	NA		
Other	4	0	0	4	0		100.00%		100.00%		NA	NA	NA		
No sexual offense/child pornography	59	0	0	58	1		98.31%	0.00%	100.00%		NA	NA	NA		
No criminal offenses	53	0	0	52	1		98.11%	0.00%	100.00%		NA	NA	NA		
**Conviction History**															
Child pornography	7	2	0	4	1	100.00%	80.00%	66.67%	100.00%	90.00%	66.67	1.50	1.25	4.83	0.44
Child sexual abuse	35	24	2	4	5	92.31%	44.44%	82.76%	66.67%	68.38%	49.43	2.02	2.72		
Sexual abuse of adults	6	0	0	6	0		100.00%		100.00%		NA	NA	NA		
Assault	3	0	0	3	0		100.00%		100.00%		NA	NA	NA		
Other	4	0	0	4	0		100.00%		100.00%		NA	NA	NA		
None	69	3	3	60	3	50.00%	95.24%	50.00%	95.24%	72.62%	45.24	2.21	2.21		
**Pedophilic Interest**															
Sexual fantasies with children	40	22	3	12	3	88.00%	80.00%	88.00%	80.00%	84.00%	68.00	1.47	1.47	1.23	0.27
No sexual fantasies with children	83	7	2	69	5	77.78%	93.24%	58.33%	97.18%	85.51%	55.52	1.80	1.41		
Acting out of sexual fantasies with minors	24	17	2	3	2	89.47%	60.00%	89.47%	60.00%	74.74%	49.47	2.02	2.02	1.17	0.28
No acting out of sexual fantasies with minors	99	12	3	78	6	80.00%	92.86%	66.67%	96.30%	86.43%	62.96	1.59	1.37		
No denial of pedophilic interest	44	27	5	9	3	84.38%	75.00%	90.00%	64.29%	79.69%	54.29	1.84	1.68	1.04	0.31
Denial of pedophilic interest	9	2	0	4	3	100.00%	57.14%	40.00%	100.00%	78.57%	40.00	2.50	1.75		
**ICD-10 Diagnosis**															
F00-F09: yes	2	0	0	1	1		50.00%	0.00%	100.00%		NA	NA	NA	3.04	0.08
F00-F09: no	122	29	5	80	8	85.29%	90.91%	78.38%	94.12%	88.10%	72.50	1.38	1.31		
F10-F19: yes	26	10	1	13	2	90.91%	86.67%	83.33%	92.86%	88.79%	76.19	1.31	1.29	0.00	0.96
F10-F19: no	98	19	4	68	7	82.61%	90.67%	73.08%	94.44%	86.64%	67.52	1.48	1.36		
F20-F29: yes	8	3	0	5	0	100.00%	100.00%	100.00%	100.00%	100.00%	100.00	1.00	1.00	1.09	0.30
F20-F29: no	116	26	5	76	9	83.87%	89.41%	74.29%	93.83%	86.64%	68.11	1.47	1.36		
F30-F39: yes	10	4	1	3	2	80.00%	60.00%	66.67%	75.00%	70.00%	41.67	2.40	2.50	3.80	0.05
F30-F39: no	114	25	4	78	7	86.21%	91.76%	78.13%	95.12%	88.99%	73.25	1.37	1.28		
F40-F49: yes	6	1	0	4	1	100.00%	80.00%	50.00%	100.00%	90.00%	50.00	2.00	1.25	0.18	0.67
F40-F49: no	118	28	5	77	8	84.85%	90.59%	77.78%	93.90%	87.72%	71.68	1.40	1.33		
F50-F59: yes	6	1	0	4	1	100.00%	80.00%	50.00%	100.00%	90.00%	50.00	2.00	1.25	0.18	0.67
F50-F59: no	118	28	5	77	8	84.85%	90.59%	77.78%	93.90%	87.72%	71.68	1.40	1.33		
F60-F69: yes	44	16	5	19	4	76.19%	82.61%	80.00%	79.17%	79.40%	59.17	1.69	1.70	5.72	**0.017**
F60-F69: no	80	13	0	62	5	100.00%	92.54%	72.22%	100.00%	96.27%	72.22	1.38	1.08		
F70-F79: yes	10	4	0	3	3	100.00%	50.00%	57.14%	100.00%	75.00%	57.14	1.75	2.00	3.80	0.05
F70-F79: no	114	25	5	78	6	83.33%	92.86%	80.65%	93.98%	88.10%	74.62	1.34	1.31		
F80-F89: yes	1	1	0	0	0	100.00%		100.00%			NA	NA	NA	0.13	0.72
F80-F89: no	123	28	5	81	9	84.85%	90.00%	75.68%	94.19%	87.42%	69.86	1.43	1.34		
F90-F99: yes	3	3	0	0	0	100.00%		100.00%			NA	NA	NA	0.39	0.53
F90-F99: no	121	26	5	81	9	83.87%	90.00%	74.29%	94.19%	86.94%	68.47	1.46	1.35		
**Pharmacological Treatment**															
Yes	32	16	3	11	2	84.21%	84.62%	88.89%	78.57%	84.41%	67.46	1.48	1.45	0.23	0.63
No	40	13	2	19	6	86.67%	76.00%	68.42%	90.48%	81.33%	58.90	1.70	1.60		
>6 Months: Yes	20	10	3	5	2	76.92%	71.43%	83.33%	62.50%	74.18%	45.83	2.18	2.07	0.24	0.63
>6 Months: No	36	11	2	18	5	84.62%	78.26%	68.75%	90.00%	81.44%	58.75	1.70	1.59		
Current: Yes	25	12	2	9	2	85.71%	81.82%	85.71%	81.82%	83.77%	67.53	1.48	1.48	0.30	0.58
Current: no	37	13	3	16	5	81.25%	76.19%	72.22%	84.21%	78.72%	56.43	1.77	1.74		
Current testosterone lowering therapy: Yes	7	5	2	0	0	71.43%		100.00%	0.00%		NA	NA	NA	1.99	0.16
Current testosterone lowering therapy: No	111	23	3	76	9	88.46%	89.41%	71.88%	96.20%	88.94%	68.08	1.47	1.28		

Performance of the best-performing model (i.e., stacked IOT+SDT model) across subgroups defined by clinical and forensic characteristics, including victim sex, hospitalization status, crime and conviction history, pedophilic interest, diagnosis according to ICD-10, and pharmacological treatment. P values and χ^2^ statistics were obtained from χ^2^-tests comparing the balanced accuracy across the depicted subgroups. Because some subgroups did not contain any positives, not all performance metrics could be calculated for every subgroup. The ICD-10 category F60-F69 does not include pedophilia (F65.4). *Abbreviations:* BAC, balanced accuracy; FN, number of false negatives; FP, number of false positives; N, number of negatives; NPV, negative predictive value; P, number of positives; PPV, positive predictive value; Sens, sensitivity; Spec, specificity; T, total number of individuals in the subgroup; TN, number of true negatives; TP, number of true positives. NA indicates that the metric was not estimable because the subgroup contained no positive or no negative cases.

## Data Availability

Since this dataset contains highly sensitive information, it will not be made publicly available upon publication. However, access to the dataset can be permitted upon direct request from the authors.

## Code Availability

The machine learning platform Neurominer is freely available as an open-source software (https://neurominer-git.github.io/NeuroMiner/intro.html). The preprocessing codes employed before the Neurominer software can be requested from the corresponding authors.
